# Cardiac Timeless Trans-Organically Regulated by miR-276 in Adipose Tissue Modulates Cardiac Function

**DOI:** 10.1093/function/zqad064

**Published:** 2023-11-27

**Authors:** Chao Tang, Qiufang Li, Xiaoya Wang, Zhengwen Yu, Xu Ping, yi Qin, Yang Liu, Lan Zheng

**Affiliations:** Key Laboratory of Physical Fitness and Exercise Rehabilitation of Hunan Province, Hunan Normal University, 410012 Changsha, China; Key Laboratory of Physical Fitness and Exercise Rehabilitation of Hunan Province, Hunan Normal University, 410012 Changsha, China; Key Laboratory of Physical Fitness and Exercise Rehabilitation of Hunan Province, Hunan Normal University, 410012 Changsha, China; Key Laboratory of Physical Fitness and Exercise Rehabilitation of Hunan Province, Hunan Normal University, 410012 Changsha, China; Key Laboratory of Physical Fitness and Exercise Rehabilitation of Hunan Province, Hunan Normal University, 410012 Changsha, China; Key Laboratory of Physical Fitness and Exercise Rehabilitation of Hunan Province, Hunan Normal University, 410012 Changsha, China; Key Laboratory of Physical Fitness and Exercise Rehabilitation of Hunan Province, Hunan Normal University, 410012 Changsha, China; Key Laboratory of Physical Fitness and Exercise Rehabilitation of Hunan Province, Hunan Normal University, 410012 Changsha, China

**Keywords:** Drosophila, heart function, miRNA276a/b, Timeless, adipose tissue, exercise-training

## Abstract

The interconnection between cardiac function and circadian rhythms is of great importance. While the role of the biological clock gene Timeless (Tim) in circadian rhythm has been extensively studied, its impact on cardiac function remains largely been unexplored. Previous research has provided experimental evidence for the regulation of the heart by adipose tissue and the targeting of miR-276a/b on Timeless. However, the extent to which adipose tissue regulates cardiac Timeless genes trans-organically through miR-276a/b, and subsequently affects cardiac function, remains uncertain. Therefore, the objective of this study was to investigate the potential trans-organ modulation of the Timeless gene in the heart by adipose tissue through miR-276a/b. We found that cardiac-specific Timeless knockdown and overexpression resulted in a significant increase in heart rate (HR) and a significant decrease in Heart period (HP), diastolic intervals (DI), systolic intervals (SI), diastolic diameter (DD), and systolic diameter (SD). miR-276b systemic knockdown resulted in a significant increase in DI, arrhythmia index (AI), and fractional shortening (FS) significantly increased and SI, DD and SD significantly decreased. Adipose tissue-specific miR-276a/b knockdown and miR-276a overexpression resulted in a significant increase in HR and a significant decrease in DI and SI, which were improved by exercise intervention. This study presents a novel finding that highlights the significance of the heart circadian clock gene Timeless in heart function. Additionally, it demonstrates that adipose tissue exerts trans-organ modulation on the expression of the heart Timeless gene via miR-276a/b.

## Graphical Abstract

**Graphical Abstract:**
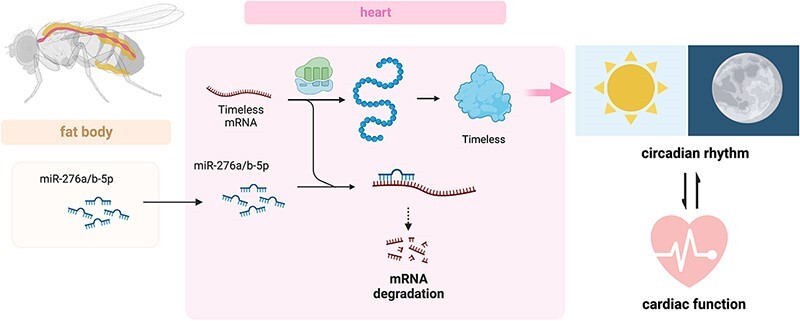

## Introduction

Cardiovascular disease (CVD) poses a huge health and economic burden worldwide. Given its role as the pumping organ of the human body, the function of the heart is vital to human health. Heart failure and arrhythmia often occur during acute and scar healing in patients with myocardial infarction, which is a significant cause of death. Yet, the pathogenesis of cardiovascular disease remains incompletely understood

Circadian rhythms are pervasive life activities in animals that occur in cycles of day and night,^1^ and at the molecular level consist of a transcription-translation feedback loop (TTFL) composed of 4 biological clock cogs, Period (Per), Timeless (Tim), CLOCK, and CYCLE.^2^ More than 13% of genes in the heart exhibit rhythmic expression.^3^ The circadian clock of heart muscle cells and all cell types associated with cardiovascular disease are recognized,^4^ and be found to regulate intrinsic properties of the heart, including its growth and remodeling, transcription, and contractile function.^5^ Circadian rhythms and sleep disorders are strongly associated with increased incidences of heart and cardiovascular disease.^6^ Epidemiologic observations also show an increase in cardiovascular disease among night shift workers.^7^ All of this suggests that changes in circadian rhythms are closely linked to heart function.

Timeless is the second biological clock gene discovered to regulate circadian rhythms through photo-regulation or by forming a heterodimer with Per that regulates circadian rhythms through positive and negative regulation.^8^ Research on other biological clock genes Per, Bmall has shown that altered expression of these genes leads to impaired cardiac contractile function,^9^ decreased heart rate (HR), and increased risk of arrhythmias,^10^ bradycardia, and a generalized hypotensive phenotype.^11^ However, the relationship between Tim and heart function has been largely unexplored, and here we innovatively explore the effect of the circadian clock gene Tim on heart function.

In recent years, adipose tissue has been identified to play an important role in regulating cardiovascular,^12^, ^13^ affecting normal/patient cardiac function,^14^, ^15^ It is also closely associated with myocardial fibrosis and atherosclerosis.^16^ Bioactive products secreted by adipose tissue include adipokine, microvesicles, and microRNAs (miRNAs).^17^ Concurrently, studies have demonstrated that adipose tissue miRNAs regulate the function of heart organs,^18^ and circulating miRNAs originate from adipose tissue.^19^ While recent studies have found that neuronal miR276a/b in the brain target Tim and thus have an important effect on sleep,^20^, ^21^ with a corresponding degree of circadian dysrhythmia,^22^ however, there are few reports on adipose tissue cross-organ regulation of cardiac Tim and thus affecting cardiac function.

Fruit flies serve as pioneering models to uncover the effects of heart development, function,^23^, ^24^ and exercise on heart health,^25–27^ and their cardiac function can be measured by M-mode.^28^ Adipose bodies in fruit flies are analogous mammalian equivalent of adipose tissue,^29^ which is important in cross-organ regulatory studies.^30^ In addition, physical activity is beneficial cardiovascular health,^31^ and a range of changes in the cardiovascular system occur at the molecular level following exercise interventions.^32–34^ Furthermore, exercise can also affect the release of miRNAs from adipose tissue,^35^, ^36^ and the role of exercise is initially explored in this paper.

## Materials and Methods

### Fly Stocks and Groups

Drosophila melanogaster were reared on a standard cornmeal/agar Drosophila diet on a 12:12 LD cycle at 25°C and 50% humidity. w^1118^ (wild-type), miR-276a^KO^ (BS58906), miR-276b^KO^ (BS50907), UAS-miR-276a (BS41143), UAS-276a/b- Sponge (BS61407), UAS-miR-276a (BS41143), UAS-miR-276b (BS41162), UAS-tim (BS80686), Hand-Gal4 (BS33832), and Cg-Gal4 (BS7011) were purchased from the Drosophila Reserve Center in Bloomington. TimRNAi (V2886) was from the Victoria Drosophila Center. Cardiomyocyte-specific expressing Drosophila and control Drosophila were generated by crossing female Hand-Gal4 with male W^1118^ or UAS-tim, timRNAi. Adipose tissue-specific expressing Drosophila and control Drosophila were generated by crossing female Cg-Gal4 with male W^1118^ or UAS-276a/b-Sponge, UAS-miR-276a, UAS-miR-Sponge. All crosses were collected within 12 h from females with F1 characteristics. All experiments used 15 d young virgin flies.

### Exercise-Training Device and Protocols

We developed a Drosophila locomotion device that takes advantage of the negative anisotropy of Drosophila to scale up by flipping the glass tube and adjusting the distance between the glass tube and sponge plug the locomotion distance at a constant rate of 8 cm. Referring to various exercise protocols and modifying them appropriately,^26^, ^49^ Drosophila flies of the long-term regular exercise group were allowed to exercise from the second day after fledging, and then rested for 2 d after exercising for 5 d, and exercised for 5 d again. Exercise was ended by 13 d. Exercise time was the same time period every day for 2.5 h. The exercise speed of the exercise device was 24 s/r. Each group of fruit flies was tested for a variety of indicators the day after the exercise intervention.

### Real-Time Quantitative PCR (qPCR)

Ten whole bodies or 40 hearts or 20 adipose tissue tissues were collected from each group of Drosophila, and total RNA was extracted from the lysates using Trizol (Invitrogen, CA, USA) reagents, according to the kit instructions. 1 μl of RT product was mixed with 10 μl of SYBR qPCR Mastermix (TaKaRa) containing the appropriate PCR primers.

qPCR amplification reactions were replicated, thermocycled, and fluoresced three times using an ABI7300 real-time PCR instrument (Applied Biosystems, USA): (30 s at 95°C, 5 s at 95°C, and 30 s at 60°C) ×40. rp49/u6 standard SYBR Green (Takara) for real-time PCR. The relative abundance of the measured genes was calculated by the 2^−ΔΔCt^ method and normalized with GAPDH. The primer used were as follows (5′-3′):

**Table:** 

U6 F:	TGGCCCCTGCGCAAGGATG
miR-276a F:	ctCAGTCAGACCCCATGTAGGAACTTC
miR-276b F:	ctCAGTCAGACCCCATGTAGGAACTTA
miRNA R:	Reverse primer from a miRcute miRNA quantitative RT-PCR detection kit (Takara)
Rp49 F:	CTAAGCTGTCGCACAAATGG
Rp49 R:	AACTTCTTGAATCCGGTGGG
Timeless (Tim) F:	CGGAGTGGTCTCAAGGTTCC
Timeless (Tim) R:	TGGATTGCTCGTTGTTCCAC

### Semi-intact Drosophila Heart Preparation and Cardiac Function Analysis

Following previous research methods,^50^ artificial hemolymph fluid was prepared and placed at room temperature to pump oxygen for 30 min. Drosophila are anesthetized with carbon dioxide and fixed to a Petri dish coated with medical petroleum jelly. Add artificial lymph. Under a stereo-microscope, the cuticles of the head, chest, and abdomen were removed with disemboweling scissors and precision forceps, the viscera was scraped away, and the accumulated fat was sucked into a microstraw to expose the heart. The HR of fruit flies (124 fps in 30 ss of video frame rate) was recorded using a scientifically developed high-sensitivity camera,^51^ HR data were collected using HC Image software, and the image data were analyzed using semi-automated optical HR analysis to obtain quantitative indicators: heart rate (HR), Heart Period(HP), diastolic intervals(DI), systolic intervals(SI), arrhythmia index (AI), diastolic diameter (DD), systolic diameter (SD) and fractional shortening (FS). The sample of each group was 20 ± 5.

### Negative Geotaxis Assay

In order to test the climbing ability of Drosophila, a Drosophila climbing ability test device was designed using the negative fast iterative negative approach of fruit flies.^52^ Drosophila was transferred to an 18-cm glass tube with an inner diameter of 2.8 cm, and Drosophila crawled along the wall of the glass tube out of instinct, and the climbing height of Drosophila was recorded when Drosophila was crawling for 8 s. The glass tube was shaken every 30 s so that the Drosophila returned to the bottom of the tube, and the process was repeated 8 times. The entire climbing process was filmed with a speed camera and we had the fruit flies acclimatized in a glass tube for 10 min before filming. The fourth, fifth, and sixth images of the climb were intercepted at the end of the tenths and the number of fruit flies reaching the top area was counted. Climbing index = number of fruit flies/total number of fruit flies in the highest area, *N* = 100.

### Western Blots

A total of 80 cardiac tubes were collected and homogenized by adding RIPA lysates (extracted from solar organisms) and protease inhibitor mixtures using key antibodies, including Anti-Tim antibody (ab133569, 1:3000, Abcam) and beta-Tubulin Ab (T0023, 1:10 000, Affinity). Secondary antibodies used included goat secondary antibody against rabbit IgG (H + L), HRP (AWS0002: 1:5000, Abiowell), and goat secondary antibody against mouse IgG (H + L), HRP (AWS0001). Densitometric analysis and quantitative evaluation were performed using ImageJ software.

### 293T Cell Culture and Luciferase Reporter Assay

Luciferase assays (Dual-Luciferase Reporter Assay System, Promega) were performed for 48 h after transfection. The full length 3’UTR of Tim was cloned into a psiCheck-2 vector (Tsingke), mutations were constructed by altering the binding site of miR-276a on the Tim 3’UTR in vitro, media used DMEM (BasalMedia).

### Statistical Analysis

The data were mapped using GraphPad Prism 9 software and Statistical Analysis using Statistical Package for (SPSS) version 25. W^1118^ and W^1118+E^ groups were compared using independent t-assays. Another inter-group analysis was used by one-way analysis of variance (ANOVA) and minimal significant difference (LSD). The significance level was set as *P* < 0.05.

## Results

### Modifying Cardiac Timeless Expression Results in Low Climbing Ability and Impaired Cardiac Function

**Figure 1.**
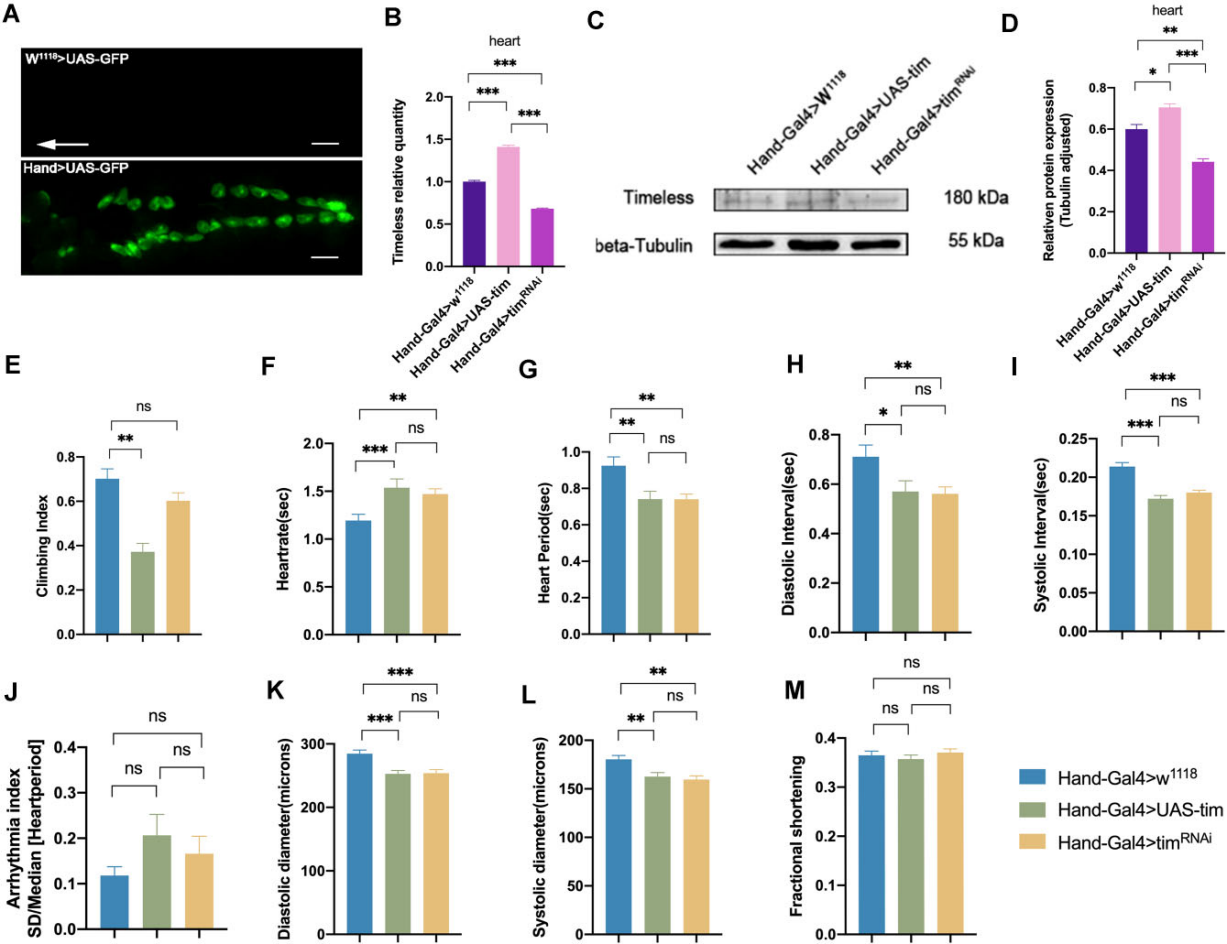
Effect of cardiac Timeless on athletic abilities and cardiac function. [Note: (A) Hand-Gal4 > UAS-GFP Drosophila fluorescence, W^1118 ^> UAS-GFP control. Arrow direction is from tail to head, scale bar = 300 µm; (B) mRNA levels of Tim in Drosophila heart; (C) protein blot analysis; (D) protein blot analysis, *n* = 80; (E) climbing ability of Drosophila, *N* = 100; (F-M) M-mode metrics of HR, HP, DI, SI, AI, DD, SD, and FS, *N* = 20. All *P*-values are from one-way ANOVA, *P < 0.05, **P < 0.01, ***P < 0.001.]

Using the Gal4/UAS transgenic system (HandGal4 > UAS-GFP) with W^1118 ^> UAS-GFP as a control, we observed Hand-Gal4 drives the myocardium gene expression (Figure 1A). The effect of Tim on cardiac function was explored using Hand-Gal4-driven knockdown and over-expression in the heart, which showed a 41% over-expression efficiency of Hand-Gal4 > UAS-tim and a 31% knockdown efficiency of Hand-Gal4 > tim^RNAi^ compared with the Hand-Gal4 > W^1118^ group (Figure 1B). Cardiac protein Western blot analysis showed a significant increase in Tim protein content in the Hand-Gal4 > UAS-tim group (*P* < 0.05) and a significant decrease in the Hand-Gal4 > tim^RNAi^ group (*P* < 0.01) (Figure 1C and D). Climbing ability was significantly reduced in the Hand-Gal4 > UAS-tim group compared to the control group (*P* < 0.01) (Figure 1E). M-mode was utilized to quantify HR, heart period (HP), SI, and DI, arrhythmia index (AI), systolic diameter (SD), diastolic diameter (DD), and Frantional Shortening (FS), and the results showed that compared with the control group, the Hand-Gal4 > UAS-tim versus Hand-Gal4 > tim^RNAi^ group HR were significantly increased (*P* < 0.01) (Figure 1F), and HP, DI, SI, DD, and SD were significantly decreased (*P* < 0.01 or *P* < 0.05) (Figure 1G, H, I, K, and L). These results suggest that elevated Tim expression in the heart significantly reduces the ability of fruit flies to exercise, while elevated or decreased Tim expression in the heart leads to impaired heart function.

### Reduced Expression of miR-276a and miR-276b Correlates With Increased Timeless Expression in the Heart and Impaired Cardiac Function

**Figure 2.**
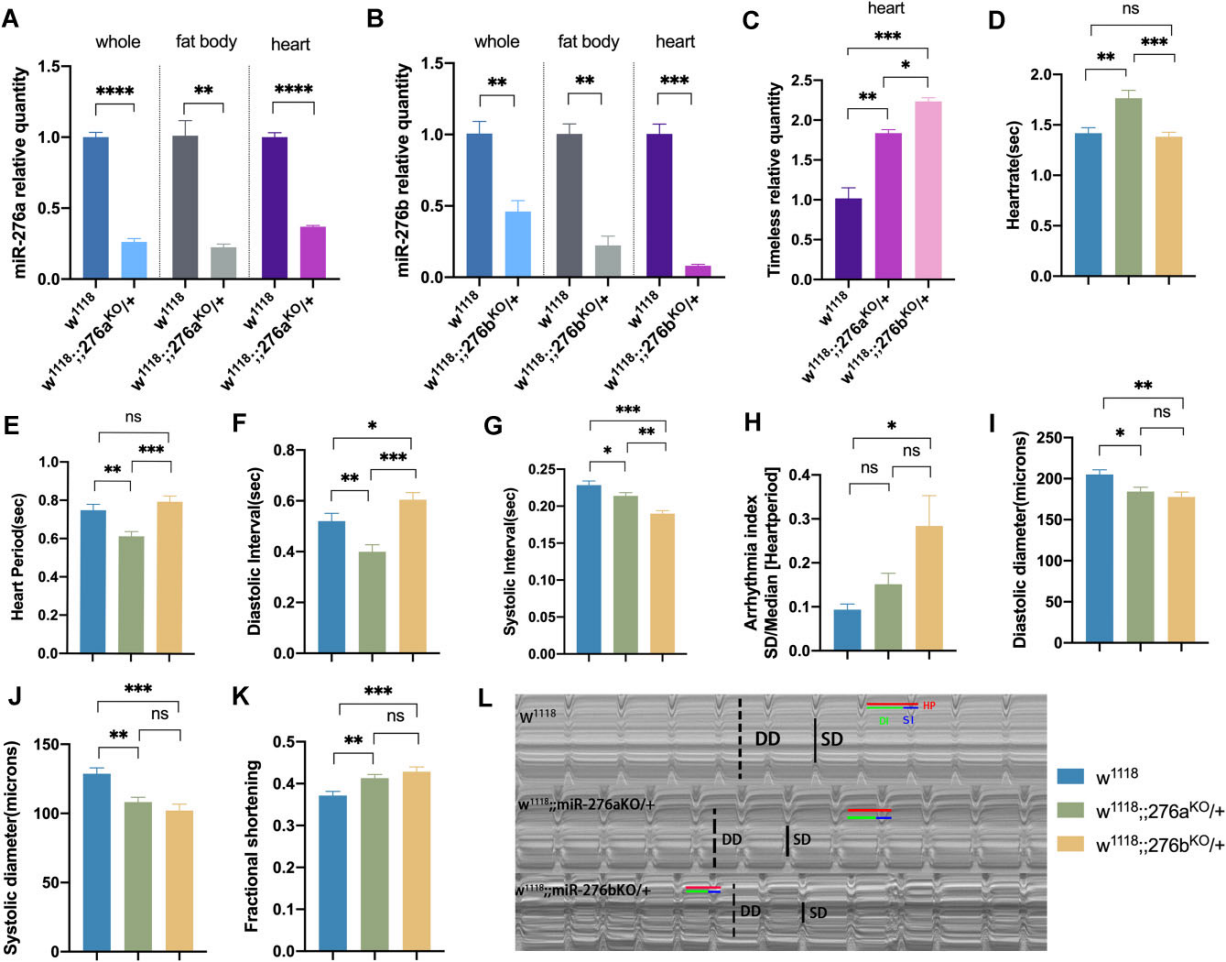
Effects of miR-276a/b on Timeless expression and cardiac function. [Note: (A) Relative expression of miR-276a in whole body, fat, and heart under miR-276a systemic knockdown condition (B) Relative expression of miR-276b in whole body, fat, and heart under miR-276b systemic knockdown condition (C) Relative expression of TIM in the heart under miR-276a, miR-276b systemic knockdown condition (2D-2K). Effect of miR-276a, miR-276b systemic knockdown on cardiac function with M-mode indexes of HR, HP, DI, SI, AI, DD, SD, and FS. *n* = 20. (L) Model M. electrocardiogram with a 10-s interception time for each group horizontal green line: DI horizontal blue line: SI, horizontal red line: HP; vertical dotted line: DD. Vertical solid lines: All *P*-values of SD were derived from one-way ANOVA, *P < 0.05, **P < 0.01, ***P < 0.001].

Gene expression levels in whole body, fat, heart, and cardiac function were measured using miR-276aKO and miR-276bKO Drosophila lines as a control group using W^1118^. The knockdown efficiency of miR-276aKO line miR-276a was 74%, 78%, and 63% for the whole body, fat, and heart, respectively (Figure 2A). miR-276bKO line had knockdown efficiency of 54%, 78%, and 99% of miR-276b, respectively (Figure 2B). In miR-276aKO and miR-276bKO, mRNA levels of Tim in the heart respectively elevated by 84% and 124% (Figure 2C). miR-276aKO showed a significant increase in HR, FS (*P* < 0.01) (Figure 2D, K) and a significant decrease in HP, DI, SI, DD, and SD (*P* < 0.01 or *P* < 0.05) (Figure 2E, F, G, I, and J). miR-276bKO increased significantly in DI, AI, FS (Figure 2G, H, and K) (*P* < 0.05 or *P* < 0.001) and decreased significantly in SI, DD, SD (*P* < 0.01 or *P* < 0.001) (Figure 2G, I, and J). These results indicated a significant decrease in miR-276a and miR-276b expression in miR276aKO and miR276bKO and a significant increase in Tim transcript levels in fruit flies. In terms of heartbeat rhythm, the knockdown of miR-276a caused the simultaneous reduction of cardiac inter-systolic and DIs, resulting in accelerated heartbeat. miR-276b knockdown caused significant arrhythmia. In terms of cardiac pumping function, both knockdowns caused shorter diastolic and systolic diameters, which led to compensatory enhancement of myocardial pumping function.

### Effects of Adipose Tissue miR-276a/B and Exercise Intervention on Cardiac Timeless

**Figure 3.**
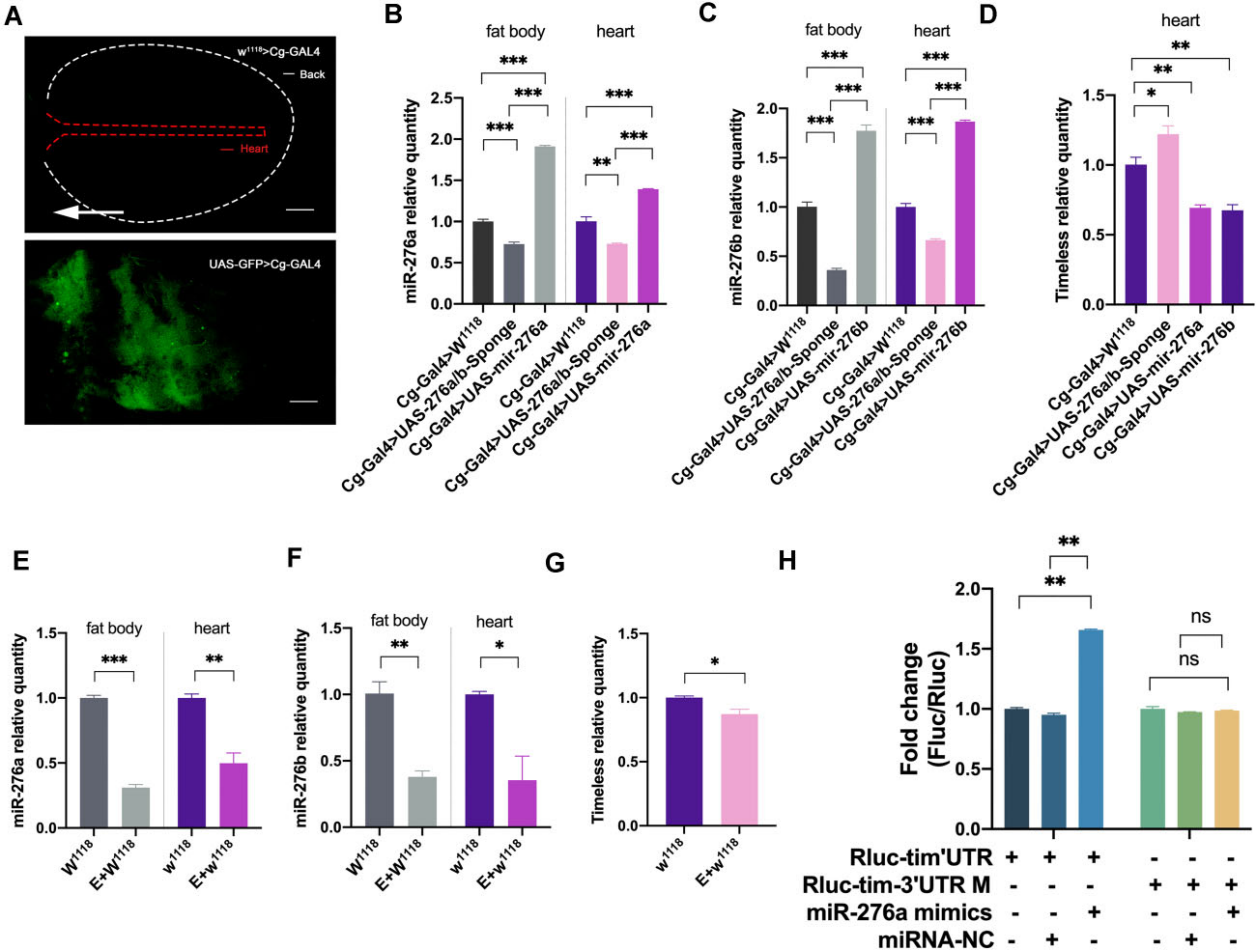
Effects of adipose tissue miR-276a/b and exercise intervention on cardiac Timeless. [Note: (A) UAS-GFP > Cg-Gal4 Fruit Fly Fluorescence, W^1118 ^> Cg-Gal4 Control, arrow direction from tail to head, white line for Drosophila dorsal plate outline, red line for Drosophila heart outline. Scale = 1 mm. (B) miR-276a in adipose tissue and heart of Cg-Gal4 > UAS-276a/b-sponge, Cg-Gal4 > UAS-miR-276a compared to Cg-Gal4 > W^1118^. (C) Levels of miR-276b in Cg-Gal4 > UAS-276a/b-Sponge, Cg-Gal4 > UAS-miR-276b adipose tissue, heart, with Cg-Gal4 > W^1118^ as control. (D) Expression levels of Timeless in cardiac Cg-Gal4 > Cg-Gal4 > UAS-276a/b-Sponge, Cg-Gal4 > UAS-miR-276a, UAS-miR-276b adipose tissues. (E) Levels of miR-276a in cardiac adipose tissue from W^1118^ exercise group, as compared to W^1118^ quiet group. (F) Levels of miR-276b in adipose tissue, the heart of W^1118^ exercise group with W^1118^ quiet group as the control group. (G) Expression levels of Timeless in the heart of W^1118^ exercise group were controlled by W^1118^ quiet group. (H) Identification of miR-276a targets. Renilla luciferase is used as a primary reporter gene, and the tim 3’UTR can be cloned into the multiple cloning region located downstream of the Renilla luciferase translational stop codon. *P*-value in the B-D group was derived from one-way ANOVA, and *P*-value in the E-H group was derived from *t*-testing of independent samples, *P < 0.05, **P < 0.01, ***P < 0.001.]

Gal4/UAS transgenic system (UAS-GFP > Cg-Gal4) using ^W1118 ^> Cg-Gal4 as control showed that Cg-Gal4 drives adipose tissue gene expression (Figure 3A). Using Cg-Gal4 with UAS-276a/b-Sponge, UAS-miR-276a, and UAS-miR-276b to drive adipose tissue-specific knockdown and over-expression, the results showed that the knockdown efficiency of miR276a in adipose tissue of the Cg-Gal4 > UAS-276a/b- Sponge line was 27% (Figure 3B), and that of miR-276b was 64% (Figure 3C), and in the heart miR-276a levels decreased by 27% (Figure 3C), miR276b levels decreased by 34% (Figure 3C), and Tim expression levels increased by 22% (Figure 3D). miR-276a over-expression efficiency in adipose tissues of the Cg-Gal4 > UAS-miR- 276a line was 91% (Figure 3B). cardiac miR-276a levels increased by 39% (Figure 3B), and Tim expression levels decreased by 31% (Figure 3D). miR-276b over-expression efficiency in adipose tissue of the Cg-Gal4 > UAS-miR-276b line was 77% (Figure 3B), cardiac miR-276a levels increased by 87% (Figure 3B), and Tim expression levels decreased by 32% (Figure 3D). Using the Drosophila exercise device developed in our laboratory for exercise intervention, with ^W1118^ as the experimental strain, miR-276a expression level in adipose tissue decreased by 69% (Figure 3E) and miR-276b decreased by 62% (Figure 3F) in the exercise group, and cardiac miR-276a expression level decreased by 50% (Figure 3E) and miR-276b decreased by 65% (Figure 3F), and cardiac Tim expression level decreased by 13% (Figure 3G). The results showed that reduction/increase adipose tissue miR-276a/b in adipose tissue led to decrease/increase of miR-276a/b in adipose tissue and heart, and increase/decrease in Tim expression in the heart, which is another indication of adipose tissue-heart cross-organ regulation in Drosophila. In wild-type Drosophila, exercise reduced miR-276a/b in adipose tissue and heart, however, cardiac Tim levels strangely did not increase, but significantly decreased. In addition, To test whether Tim can be actually regulated by miR-276a, 293T cells were transfected with plasmids expressing Tim 3’UTR connected downstream from a Renilla luciferase reporter. The results showed that miR-276a increased fluorescent expression by 65.7%, which could be rescued by mutation of the Tim 3’UTR binding site (Figure 3H).

### Effects of Adipose Tissue miR-276a/B and Exercise Intervention on Athletic Abilities and Cardiac Function

**Figure 4.**
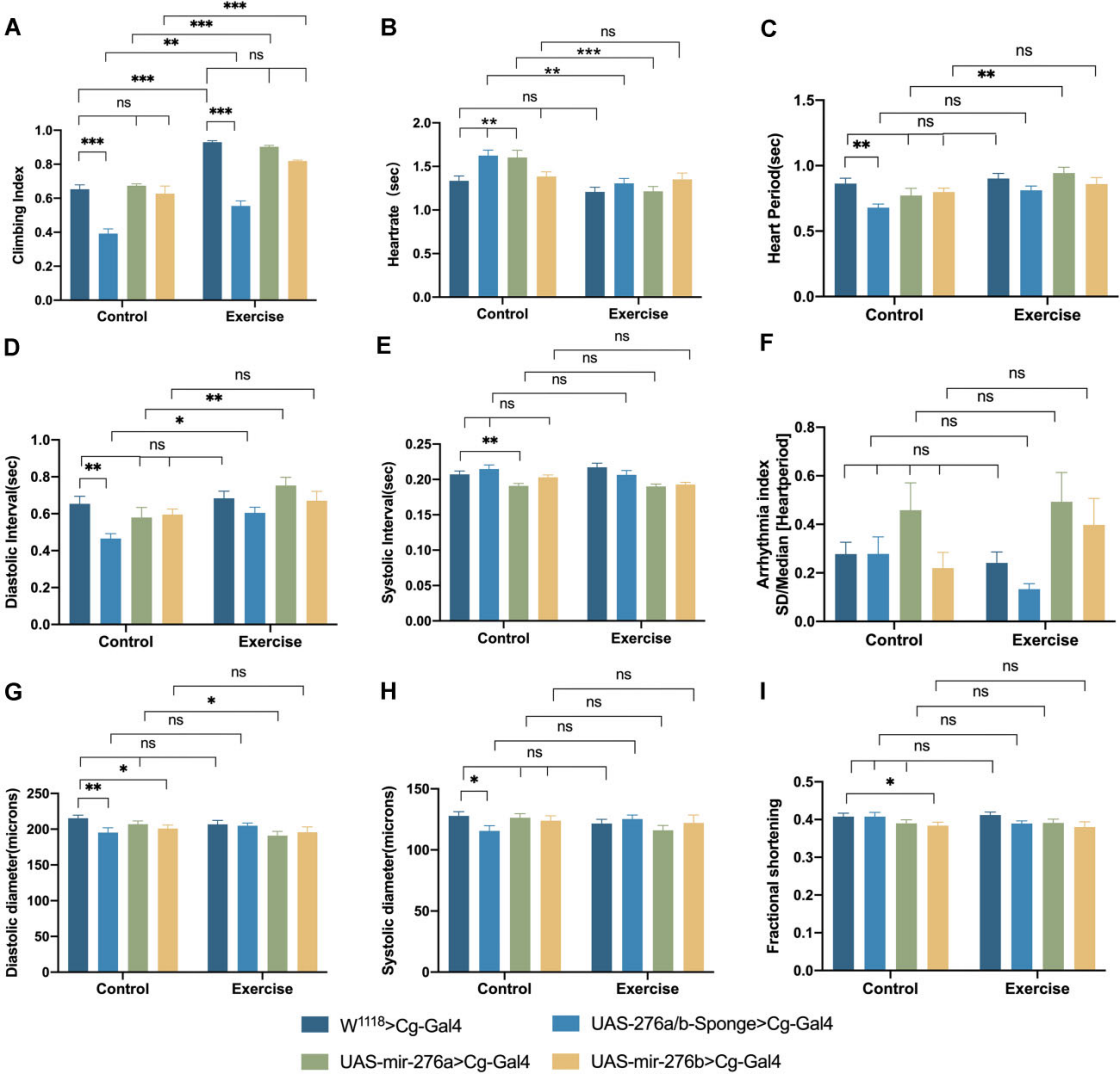
Effects of adipose tissue miR-276a/b and exercise intervention on athletic abilities and heart function. [Note: (A) Effects of knockdown, over-expression of miR-276a/b in adipose tissue and exercise intervention on Drosophila athletic ability, *N* = 100. (B-I) Effects of adipose tissue miR-276a/b knockout, over-expression, and exercise intervention on cardiac function, with M-mode indicators being HR, HP, DI, SI, AI, DD, SD, and FS. *N* = 20. All *P*-values are from one-way ANOVA. Values are from one-way ANOVA. *P < 0.05, **P < 0.01, ***P < 0.001]

The climbing index was significantly lower in the Cg-Gal4 > UAS-276a/b-Sponge group compared with Cg-Gal4 > W^1118^ in each group within the group (*P* < 0.001), and there was no significant difference in the over-expression group (*P *> 0.05), the climbing index was significantly higher in each of the long-term regular exercise groups between the groups compared with the control group, respectively (*P* < 0.01 or *P* < 0.001) (Figure 4A), indicating that decreased miR-276a/b levels led to decreased exercise capacity, however, long-term regular exercise improved the decreased exercise capacity caused by decreased miR-276a/b levels. In terms of cardiac function, the HR in the control group Cg-Gal4 > UAS-276a/b-Sponge and Cg-Gal4 > UAS- miR-276a was significantly increased compared to Cg-Gal4 > W^1118^(*P* < 0.01 or *P* < 0.001), and the HR was significantly reduced in the long-term routine exercise group UAS-miR-276a > UAS-miR-276b compared to the control group (Figure 4B). The cardiac cycle, as the reciprocal of HR, showed a trend opposite to HR (Figure 4C). The cardiac cycle included SI and DI, and in the Cg-Gal4 > UAS-276a/b-Sponge and Cg-Gal4 > UAS-miR-276a groups, the HR increased due to DI and SI, respectively (*P* < 0.01). The ameliorative effect on HR from exercise was mainly due to longer DI (Figure 4D, E). There was no significant difference in the arrhythmia index between the two groups (*P* > 0.05) (Figure 4F). Compared to Cg-Gal4 > W^1118^, the DD decreased in the Cg-Gal4 > UAS-miR-276b group (*P* < 0.05) (Figure 4G) and FS decreased (*P* < 0.05) (Figure 4I), while the DD and SD decreased in the Cg-Gal4 > UAS-276a/b-Sponge group (*P* < 0.01 or *P* < 0.05) (Figure 4G).

## Discussion

The identification of the circadian rhythm gene has significantly enhanced the comprehension of circadian rhythms across various organisms, encompassing insects, mammals, and humans. In humans, the discovery of over a dozen circadian clock genes has shed light on the intricate mechanisms underlying circadian rhythms. Notably, a transcription-translation feedback loop (TTFL) comprising four circadian gears (Per, Tim, CLOCK, and CYCLE) assumes a crucial role in the regulation of circadian rhythms in fruit flies.^2^ Prior investigations on additional circadian clock genes have revealed their significant impact on cardiac function. Our findings indicate that the presence of PER2S662G and PER2S662D transgenic mice, which alter circadian rhythms, resulted in the inhibition of cardiac function and a reduction in FS.^9^ Additionally, Bmal1 knockout mice displayed dilated heart disease,^37^ while cardiomyocytes with Bmal1-specific knockout experienced cardiac systolic dysfunction,^38^, ^39^ a decrease in HR, and an increased risk of arrhythmia.^10^ Furthermore, Yu’s study demonstrated that the specific knockout of Bmal1 in the left stellate ganglion effectively prevented ventricular arrhythmias following myocardial ischemia.^40^ Another study revealed that *Bmal1*^−/−^ mice exhibited bradycardia and a generalized hypotensive phenotype.^11^ Regrettably, there exists a dearth of literature pertaining to the correlation between the circadian clock gene Tim and its impact on cardiac function. This may be related to the fact that Tim transgenic mice are technically difficult to realize.

Based on the aforementioned analyses, we have formulated a scientific hypothesis positing that the circadian clock gene Tim plays a regulatory role in cardiac function. To test this hypothesis, our research team employed the Hand-Gal4 technique to induce knockdown and overexpression of cardiac Tim. The findings depicted in Figure 1 demonstrate that both heightened and diminished expression of cardiac Tim result in an increased HR in Drosophila, accompanied by a decrease in diastolic duration (DD) and impaired cardiac function to a certain degree. These results indicate that deviations from the normal state of Tim gene expression can exert an influence on cardiac function. There was no statistically significant disparity observed in the impact of various circadian clock genes on AI or FS in comparison to their impact on circadian clock genes on heart function, whereas Per and Bmal1 exhibited a more pronounced influence on both parameters. These findings imply that Tim and other circadian clock genes exhibit comparable yet not identical effects on cardiac function.

Following the identification of Tim’ influence on cardiac function, our research focused on elucidating the mechanisms of miRNA regulation upstream of this gene followed by an exploration of mechanisms involved in subsequent studies regarding up-down regulation. In the initial investigation, a cohort of researchers successfully identified miRNAs that specifically target the RNA endonuclease Drosha, thereby providing confirmation of the regulatory functions exhibited by miR-276.^41^ Subsequent studies have documented the removal miR-276a/b binding sites from Tim binding sites, as well as alterations in Tim levels resulting from modified miR-276a expression in the brain. These changes in Tim levels have been observed to significantly impact sleep patterns, leading to circadian dysrhythmia.^20–22^ Our research team conducted a comprehensive bioinformatics analysis, which revealed the presence of a distinct miR-276a/b seed sequence within the 3’UTR of Tim (Supplementary Figure S1). Collectively, these reports and analyses strongly suggest a potential association between miR-276a/b and Tim, underscoring their relevance in the context of sleep regulation and circadian rhythm disturbances.

Secondly, adipose tissue, being a crucial organ in the organism, adipose tissue releases hormones and miRs either freely or through vesicle pathways. It has been widely acknowledged for its inter-organ role in the regulation of other tissue functions.^42^, ^43^ Furthermore, recent research has highlighted the significance of certain miRNAs in adipose tissue in the regulation of cardiac function. For instance, Fang et al.’s investigation revealed that miR-200a in adipose tissue exosomes targets cardiomyocytes, resulting in cardiac hypertrophy.^44^ Similarly, miR-320d in adipose tissue inhibits cardiomyocyte apoptosis associated with atrial fibrillation.^45^ Additionally, Hao’s research revealed a correlation between adipose tissue-derived miR-134-5p and remodeling following myocardial infarction.^46^ Building upon this information, our team aims to further investigate the scientific hypothesis that adipose tissue miR276a/b trans-organically modulates the expression of the Tim gene in the heart, consequently impacting cardiac function.

To validate this hypothesis, we employed a systematic knockout approach for miR-276a/b and conducted specific knockout and over-expression experiments for miR-276a or miR-276b in adipose tissue, assessing their effects on Tim gene expression and cardiac function. Figure 2 illustrates that the elevation of cardiac Tim levels resulting from systemic miR-276a and miR-276b decreases leads to a reduction in both DD and SD. The diminished DD indicates a certain degree of impaired cardiac function, while the shortened SD may be associated with the compensatory augmentation of cardiac pumping function. Specifically, the impairment of cardiac function induced by the knockdown of miR-276a or miR-276b is primarily manifested as an increase in HR or the occurrence of cardiac arrhythmia, respectively. Figure 3 and 4 demonstrate that the alteration of adipose tissue miR-276a/b levels leads to corresponding changes in adipose tissue and cardiac miR-276a/b expression, as well as cardiac Tim expression. These findings strongly suggest a partial origin of that cardiac miR-276a/b from adipose. We reviewed the literature and guessed that miR-276 is transported from adipose tissue to the heart via exosomes as carriers, but it has not been proved more directly, and we will further investigate this part in the future. Manipulation of adipose tissue miR-276a/b, such as knockout or over-expression, results in increased HR. Furthermore, knockdown of adipose tissue miR-276a/b leads to shorter diastolic duration and systolic duration, thereby impairing cardiac pumping function. Conversely, over-expression of miR-276b in adipose tissue leads to a reduction in DD and FS.

Based on the aforementioned findings, it is evident that adipose tissue miR-276a/b exerts a substantial direct influence on the levels of cardiac miR-276a/b and Tim. This observation underscores the regulatory role of adipose tissue in modulating the expression of the Tim gene in the heart, thereby impacting cardiac function through miR-276a/b. Furthermore, the alterations observed in the systemic knockout of miR-276a align closely with those observed in the specific knockout of miR-276a/b in adipose tissue, both of which result in changes in cardiac Tim levels and cardiac function. These findings suggest that adipose tissue exerts trans-organ regulation on cardiac Tim in the heart, thereby influencing cardiac function, mainly through miR-276a, not miR276b.

Previous research has demonstrated that physical activity enhances cardiovascular function, myocardial structure, and behavioral aspects (such as sleep patterns and negative emotions) in Drosophila melanogaster.^47^, ^48^ Furthermore, exercise exerts a notable influence on the release of miR within adipose tissue.^35^, ^36^ For instance, a study conducted by Kristensen et al. revealed that a combination of exercise and dietary modifications over a 15-wk period resulted in the upregulation of miR-29a-3p and miR-29a-5p, while downregulating of miR-20b-5p in subcutaneous adipose tissue during weight loss.^36^ Therefore, our study aimed to examine the potential impact of exercise on the inter-organ regulation of miR276a/b in adipose tissue, specifically focusing on its influence on Tim expression and cardiac function in the heart. Notably, our findings, as depicted in Figure 3, demonstrated a significant reduction in the expression levels of both miR-276a/b and Tim gene in adipose tissue and the heart following exercise. We attribute this to the complexity of the way in which exercise regulates gene expression in the body under normal physiological conditions, and Tim is a time-clock-critical gene that is influenced by multiple factors. We believe that this phenomenon requires more in-depth studies in the future to elucidate its mechanisms. Furthermore, the findings depicted in Figure 4 indicate that a decline in miR-276a/b levels resulted in a decrease in athletic performance. However, engaging in regular exercise over an extended period of time has the potential to ameliorate the diminished athletic ability caused by reduced miR-276a/b levels. The enduring impacts of consistent physical activity on cardiac function in fruit flies primarily involve a reduction in HR, while the pumping function remains unaffected. Preliminary evidence suggests that exercise might regulate miR-276a/b levels in the adipose tissue of fruit flies, thereby enhancing athletic performance and reducing HR during exercise. Despite the absence of elevated Tim gene expression in the heart as a result of diminished miR-276a/b levels induced by exercise, this intriguing discovery warrants further investigation in subsequent research endeavors.

## Supplementary Material

zqad064_Supplemental_File

## Data Availability

The raw data supporting the conclusions of this article will be made available by the authors, without undue reservation.
