# Supplementary material for: Cardiac Timeless Trans-Organically Regulated by miR-276 in Adipose Tissue Modulates Cardiac Function
Source: Function (Oxf). 2023 Nov 27;5(1):zqad064. doi: 10.1093/function/zqad064 (PMC10696634; doi:10.1093/function/zqad064)
Supplement: zqad064_Supplemental_File [file zqad064_supplemental_file.docx]

**Supplementary Materials**


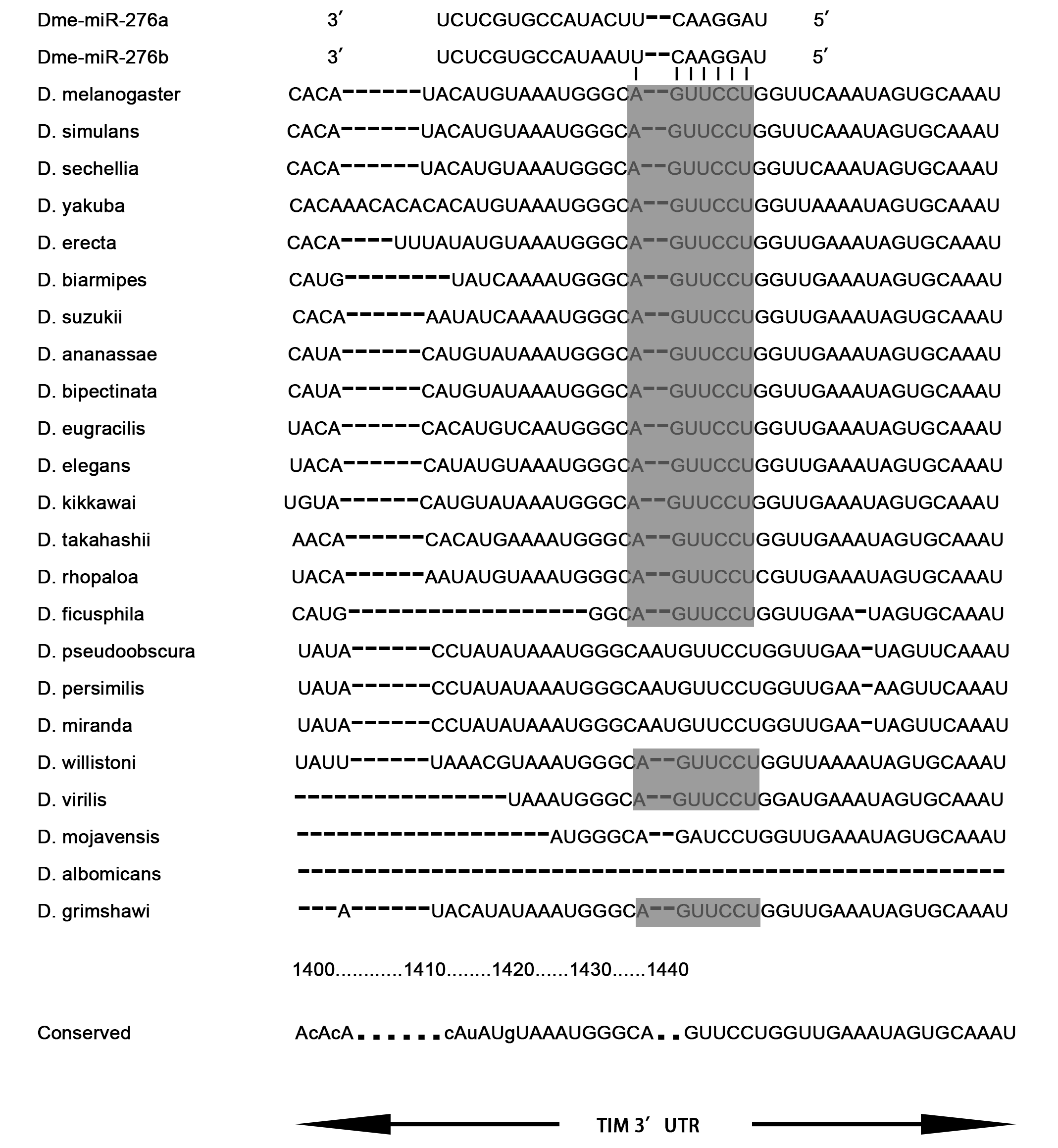


Figure S1 Bioinformatics prediction of miR-276a/b target genes

(Note: TargetScan prediction of miR-276a/b highly conserved binding sites in TIM 3 'UTR dark boxes: 18 different fruit flies from TIM 3'-UTR sequence.)
